# GPCR-bigrams: Enigmatic signaling components in oomycetes

**DOI:** 10.1371/journal.ppat.1007064

**Published:** 2018-07-05

**Authors:** Johan van den Hoogen, Francine Govers

**Affiliations:** Laboratory of Phytopathology, Wageningen University, Wageningen, the Netherlands; THE SAINSBURY LABORATORY, UNITED KINGDOM

Oomycetes are diverse eukaryotic microorganisms, comprising important pathogens as well as free-living saprophytes [1]. Plant pathogenic oomycetes cause enormous yield losses in crop plants but also threaten natural vegetation. *Phytophthora infestans*, the causal agent of potato late blight, is without a doubt the most (in)famous. Other important plant pathogenic oomycetes include downy mildews and *Albugo* [2]. Animal pathogenic oomycetes (such as *Saprolegnia*, *Aphanomyces*, and *Pythium*) primarily affect marine organisms such as fish and crustaceans but can also cause harmful diseases in humans and cattle. Here, we outline unique features of oomycetes and their cellular signaling systems. We focus on one particularly interesting class of signaling components, the so-called GPCR-bigrams, in which a typical signaling domain is preceded by a G-protein coupled receptor (GPCR) domain and propose future directions to elucidate the function and activity of this exceptional group of signaling proteins.

## What makes oomycetes exceptional?

Oomycetes are easily mistaken for fungi but are different in many ways. At the organismal level, morphology, growth pattern, and mode of dispersal are shared, and the 2 groups occupy similar ecological niches. Only at the cellular and molecular level do the differences become apparent. For example, the cell walls have a different composition; whereas chitin is the main component in fungi, oomycete cell walls consist of cellulose and β-glucans [3]. Opposed to the flattened mitochondrial cristae in fungi, oomycete mitochondria have tubular cristae [4]. Also, the actin cytoskeleton has unique features such as so-called actin plaques. These dot-like structures resemble actin patches in fungi, but unlike patches, plaques have a long lifetime and have presumably no role in endocytosis [5]. Oomycetes also exhibit distinct features in their protein repertoires, particularly with respect to protein domain organization. The number of proteins with distinct protein domain combinations is significantly higher than in other eukaryotes, and many of these, including the GPCR-bigrams, are potentially involved in cellular signaling [6].

Obviously, these differences also have consequences for the efficacy of chemical control agents. For example, a major class of fungicides broadly used in healthcare and agriculture are sterol biosynthesis inhibitors (SBIs), but since oomycetes do not synthesize sterols, they are insensitive to SBIs. The quest for novel compounds for controlling pathogenic oomycetes is a continuous challenge. We postulate that oomycete-specific cellular signaling components, such as GPCR-bigrams, hold potential as novel drug targets. Likewise, due to their central role in development and pathogenicity, fungal GPCRs have recently been proposed to be druggable targets for novel antifungal agents [7].

## What are unique features of oomycete cellular signaling?

Cellular signaling in oomycetes is inscrutable and holds many novelties. Several oomycete signaling components are clearly distinct from their homologs in organisms from other taxa. Many of these are composed of unique combinations of protein domains not known to exist in other organisms [8, 9]. For example, *Phytophthora* has several protein kinases and phospholipid kinases with accessory domains that are normally not found in combination with kinase domains. On the other hand, 2 ubiquitous enzymes, i.e., protein kinase C (PKC) and phospholipase C, seem to be absent, at least in their stereotypical forms [8, 9].

**Fig 1:**
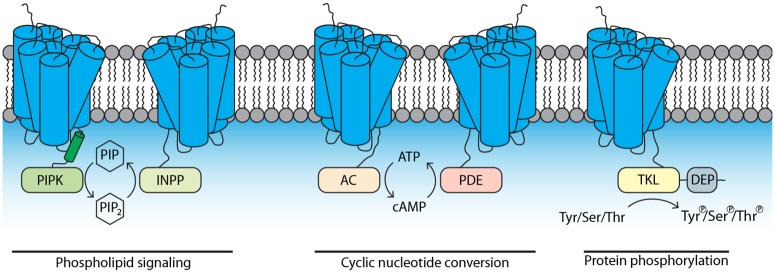
Membrane topology of GPCR-bigrams. GPCR-bigram types with a C-terminal catalytic domain predicted to be intracellular. One type not shown here is AP-GPCR, a GPCR-bigram with an N-terminal aspartic protease domain [10]. Modified from [10]. AC, adenylyl cyclase; DEP, Dishevelled, Egl-10, and Pleckstrin; GPCR, G-protein coupled receptor; INPP, inositol polyphosphate phosphatase; PDE, phosphodiesterase; PIPK, phosphatidylinositol-4-phosphate 5-kinase; PIP, phosphatidylinositol phosphate; TKL, tyrosine kinase-like.

One particularly interesting class of unique signaling components comprises the GPCR-bigrams. These proteins have an N-terminal GPCR domain typically composed of 7 transmembrane (TM) regions, combined with a C-terminal catalytic accessory domain [10]. Based on the predicted biochemical activity of the accessory domain, we anticipate that these GPCR-bigrams have roles in phospholipid signaling (GPCR-PIPKs [phosphatidylinositol-4-phosphate 5-kinase], GPCR-INPPs [inositol polyphosphate phosphatase]), cyclic nucleotide conversion (GPCR-ACs [adenylyl cyclase], GPCR-PDEs [phosphodiesterase]), or protein phosphorylation (GPCR-TKLs [tyrosine kinase-like]) (Fig 1). Most oomycetes have comparable copy numbers of the different GPCR-bigram types, with some types having only a few copies, while others belong to gene families with up to 20 members [10]. All types of GPCR-bigrams are shared by oomycetes, but some types are sparsely present in organisms from other taxa. For example, GPCR-PIPKs are found in a diverse but limited range of eukaryotic microorganisms distributed over nearly all eukaryotic supergroups [10].

The predicted catalytic activity of these GPCR-bigrams is unique. There are other examples of GPCRs with accessory domains, such as adhesion GPCRs, but their extracellular N-terminal extensions have a role in protein–protein interaction and are not predicted to have catalytic activity [11]. Plants possess regulator of G-protein signaling (RGS) proteins that, similar to GPCR-bigrams, have an N-terminal 7TM receptor domain [12]. RGS domains, however, are not catalytically active but rather accelerate the intrinsic GTPase activity of Gα subunits [12].

In oomycetes, GPCR-bigrams occur next to regular conserved enzymes. For example, *P*. *infestans* has 4 GPCR-ACs in addition to 8 canonical adenylate cyclases (ACs) (http://fungidb.org/fungidb/app/record/organism/NCBITAXON_403677). This underscores the importance of GPCR-bigrams for oomycetes. In case the catalytic domain in a GPCR-bigram would not have an advantage over the canonical enzyme, there would be no evolutionary pressure for the GPCR-bigram to sustain. Thus, the strong conservation of GPCR-bigrams throughout oomycetes indicates that having GPCR-bigrams is advantageous. Clearly, oomycetes need GPCR-bigrams, but for what purpose?

## What is known about GPCR-bigrams?

The conservation of all GPCR-bigram types in oomycetes and the presence of GPCR-PIPKs in several unicellular eukaryotes almost indisputably suggests that they are functional proteins. To date, however, there are only a few studies addressing their biological role, and these are essentially limited to GPCR-PIPKs. Knockout lines of the single GPCR-PIPK gene *RpkA* in the slime mold *Dictyostelium discoideum* displayed defects in cell density sensing, bacterial defense, and phagocytosis and had reduced phospholipid levels [13, 14]. Silencing or overexpression of 1 of the 12 GPCR-PIPK genes in *P*. *infestans* resulted in aberrant asexual development and reduced pathogenicity [15]. *Phytophthora sojae* transformants with a silenced GPCR-PIPK gene showed similar phenotypes but, in addition, showed reduced chemotaxis toward soybean root tips and the soybean isoflavone daidzein [16]. These limited experimental data show that GPCR-PIPKs are important for proper functioning of oomycetes but many questions remain. Are the accessory domains catalytically active? Are GPCR-bigrams capable of sensing a ligand? How is their activity regulated? And what is the mode of action of GPCR-bigrams?

## How do GPCR-bigrams work?

The catalytic domains of GPCR-bigrams are usually the core domains in effector proteins regulated by G-protein signaling. Hence, it is conceivable that GPCR-bigrams provide a direct link between GPCR sensing and catalytic activity. Below, we speculate how GPCR-bigrams may transduce signals.

**Fig 2:**
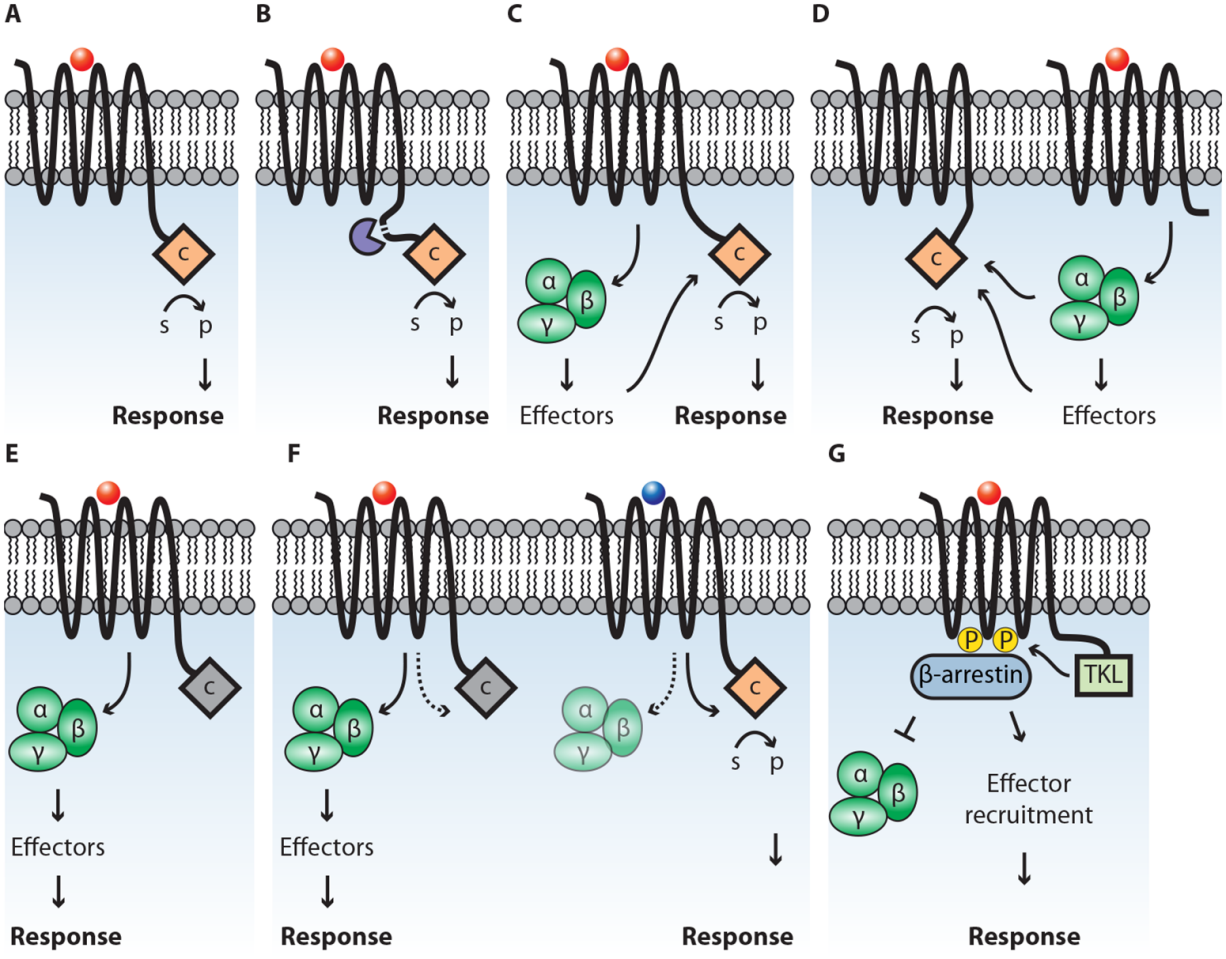
Proposed models for the mode of action of GPCR-bigrams. In each model, agonist binding on the receptor domain leads to downstream responses. In (A), the catalytic domain (c) is directly activated, leading to conversion of a substrate (s) to a product (p). In (B), proteolytic cleavage (purple) yields a mature GPCR and an active catalytic domain. In (C), G-proteins are activated, which either directly or indirectly activate the catalytic domain. In (D), the catalytic domain is activated by G-proteins or effector proteins, activated by a canonical GPCR. In (E), the catalytic domain is inactive (grey), and instead, G-proteins are activated to induce the production of second messengers. In (F), the receptor displays biased agonism and either activates G-proteins (left) or the catalytic domain (right). In (G), phosphorylation of the GPCR (yellow circles) by kinase activity of GPCR-TKLs leads to recruitment of β-arrestin, thereby either blocking signaling via G-proteins (left) or scaffolding effector proteins to initiate downstream signaling (right). GPCR, G-protein coupled receptor; TKL, tyrosine kinase-like.

An intriguing possibility is that the catalytic domain is activated directly upon binding of an agonist (i.e., a stimulating ligand) to the GPCR domain (Fig 2A) or after proteolytic cleavage (Fig 2B), thereby bypassing intermediate signaling components. Such a direct signal transfer is unprecedented and could be more efficient than via G-proteins. The downside, however, is that only a single downstream effector protein is activated. This is in contrast to a canonical GPCR that can activate multiple and different downstream effectors at once. Despite being more efficient, the direct activation may limit the signaling system in both amplitude and versatility.

Another possibility is that the GPCR domain activates heterotrimeric G-proteins that then stimulate the activity of the catalytic domain (Fig 2C). Likewise, the activation could be initiated through a second canonical GPCR (Fig 2D). Possibly, this requires dimerization of GPCR domains (not depicted). In case the catalytic domain is nonfunctional or inactive, the GPCR-bigram might act as a stereotypical GPCR, activating effector proteins via G-proteins (Fig 2E).

GPCRs can display biased agonism, a phenomenon signifying that different ligands can induce specific receptor profiles on the same receptor molecule [17]. Such profiles, represented by the receptor conformation or phosphorylation pattern, result in different downstream responses by activation of different effectors [18]. Likewise, GPCR-bigrams could show a bias toward a specific agonist, either activating G-proteins or the catalytic domain (Fig 2F).

Besides heterotrimeric G-proteins, β-arrestins can also act as molecular switches transmitting GPCR-sensed signals. Initially, β-arrestins were thought to serve a main role in the desensitization of GPCRs, initiating the internalization of an activated GPCR. Later, it was recognized that β-arrestins can facilitate signal transduction to mitogen activated protein kinases (MAPKs) by serving as scaffolds to recruit proteins to an activated GPCR [19]. The phosphorylation pattern of the receptor functions as a “barcode”, recruiting different effector proteins to β-arrestins, thereby activating distinct signaling pathways. Phosphorylation of GPCRs is typically performed by GPCR-kinases (GRKs), protein kinase A (PKA), or PKC [19], all kinases that are underrepresented in oomycetes. *P*. *infestans* has only a single GRK gene and lacks PKC [9]. It is conceivable that GPCR-TKLs have the capacity to phosphorylate GPCRs, thereby compensating for the apparent deficiency of GPCR-phosphorylating kinases. This phosphorylation can lead to β-arrestin–initiated desensitization of G-protein–mediated signaling or to recruitment of downstream effectors, such as MAPKs (Fig 2G). Similarly, GPCR-TKLs could phosphorylate another GPCR or GPCR-bigram to elicit a similar response (not depicted). Yet another possibility is that the single GRK in *P*. *infestans* phosphorylates GPCR-bigrams to initiate β-arrestin recruitment.

## How can ligands of GPCR-bigrams be identified?

Most if not all GPCRs are activated upon recognition of an external signal. Likely, the receptor domains of GPCR-bigrams are also capable of recognizing a ligand, and obvious questions that arise are the following: what is the nature of these ligands and how can they be identified? So far, the only putative candidate is the isoflavone daidzein [16]. There is no evidence, though, that daidzein is the ligand that physically interacts with the GPCR-PIPK.

As GPCRs are important drug targets in human medicine, ligand discovery is primarily focused on human GPCRs. A common and popular approach is screening (human) cells expressing the GPCR of interest with chemical libraries and monitoring changes in production of second messengers such as cAMP, IP3, or Ca^2+^ using biosensors or induction of reporter expression [20]. As yet, no secondary messenger biosensors are available for use in oomycetes. Nevertheless, some of these “reverse pharmacology” approaches might be amendable for studying GPCR-bigrams.

Though the setup would be artificial, one could envision expressing an oomycete GPCR-bigram in a mammalian cell line and then screening a chemical library of known compounds or mixtures comprising putative ligands, such as exudates from plant tissue or from *Phytophthora*. Alternatively, the GPCR-bigrams could be expressed in yeast, taking advantage of the many available signaling mutants and reporter strains. Also, yeast is easy to manipulate and contains only 2 endogenous GPCR-signaling systems that can be eliminated [21]. Of the 5 types of GPCR-bigrams, GPCR-ACs and GPCR-PDEs seem the most straightforward to tackle. By screening a chemical library for induction of cAMP production in yeast expressing a GPCR-AC, one could determine whether the AC domain is active and, if so, is regulated via the GPCR domain. To rule out that the GPCR domain can activate endogenous signaling leading to second messenger production, a truncated protein lacking the catalytic domain could be used as a control.

As yet, oomycete β-arrestins have not been studied, but given the presence of 3 proteins with arrestin domains in *P*. *infestans* (http://fungidb.org/fungidb/app/record/organism/NCBITAXON_403677), we assume that arrestins have a role in oomycete cellular signaling somehow. For analyzing β-arrestin desensitization and recruitment by GPCRs, several assays are available. Some are based on human arrestin fused to a protein that upon activation induces reporter gene expression (e.g., Tango GPCR assay system) or β-galactosidase activity (e.g., PathHunter arrestin assay) [20, 22]. Other assays make use of bioluminescence resonance energy transfer (BRET) [20], for which the GPCR-bigram has to be tagged with a fluorescent acceptor protein (e.g., GFP) and the β-arrestin with luciferase (e.g., Rluc). When in close proximity, a detectable fluorescent signal is emitted.

With the recent development of a CRISPR/Cas9 system in *Phytophthora sojae* [23], it might be achievable to create transgenic lines to study β-arrestin recruitment to GPCR-bigrams using BRET or to monitor secondary messenger production using biosensors or reporter gene expression. By targeted mutagenesis, mutants can be generated to study the role of individual domains, e.g., by removing the GPCR domain of a GPCR-bigram of interest and analyzing changes in catalytic activity. Moreover, CRISPR/Cas systems could be used to create knockouts of multiple members of 1 gene family at once, thereby avoiding redundancy.

## What lies ahead?

Our current understanding of how GPCR-bigrams function is very limited. The challenges that lie ahead are determining their role in cellular signaling and their biochemical mode of action and answering the question why oomycetes have such unique signaling proteins. What is the advantage of having GPCR domains linked to catalytic accessory domains? Does it provide, for example, shortcuts for more efficient signaling? This could be the case if the catalytic domain is under direct control of the GPCR domain, a situation that is unprecedented. Another major challenge is to identify ligands recognized by GPCR-bigrams and to determine how such ligands can be exploited. We envision that profound knowledge of these enigmatic signaling components and their ligands exposes new strategies for designing novel, oomycete-specific control agents to mitigate damage caused by these devastating pathogens.
